# A call for caution regarding infection‐acquired COVID‐19 immunity: The potentially unintended effects of “immunity passports” and how to mitigate them

**DOI:** 10.1111/jasp.12779

**Published:** 2021-05-29

**Authors:** Ricky Green, Mikey Biddlestone, Karen M. Douglas

**Affiliations:** ^1^ School of Psychology University of Kent Canterbury UK

## Abstract

Research suggests that emerging information about infection‐acquired COVID‐19 immunity should be interpreted with caution. The introduction of “immunity passports” that would enable people who have recovered from COVID‐19 to travel freely and return to work may therefore have detrimental consequences if not managed carefully. In two studies, we examined how perceived (suspected or imagined) recovery from COVID‐19, and the concept of immunity passports, influence people’s intentions to engage in behaviors aimed to reduce the spread of COVID‐19. We also consider ways to lessen potential negative effects. In Study 1 (*N* = 1604), participants asked to imagine that they had recovered from COVID‐19 reported lower social distancing intentions compared to a control condition. Participants who suspected (versus imagined) that they had recovered from past infection did not report lower preventative intentions compared to the control condition, even at high levels of certainty of past infection. In Study 2 (*N* = 1732), introducing the idea of immunity passports also reduced social distancing intentions compared to a control condition. The latter effect was, however, attenuated when cautious information about the equivocal science on COVID‐19 was also presented to participants. Participants who suspected that they had COVID‐19 in the past (compared to the control condition) revealed a similar pattern of results, but only at higher levels of certainty of past infection. Caution regarding infection‐acquired COVID‐19 immunity and immunity passports will be crucial in the COVID‐19 response. Implications for premature pandemic announcements, as well as their potential remedies, are discussed.

## 1 INTRODUCTION

“A total and complete sign off [COVID‐19] from White House Doctors yesterday. That means I can’t get it (immune), and can’t give it. Very nice to know!!!”

– Tweeted by Donald J. Trump, 11/10/2020.^1^

Governments around the world have suggested issuing “immunity passports” to people who have recovered from COVID‐19.^2^ These are certificates of sorts that would enable people to travel freely and return to the workplace under the assumption that their recovery will protect them against reinfection (McMillan, 2020). This concept, however, is at odds with some scientific reports on infection‐acquired COVID‐19 immunity, which conclude that there is currently no evidence that people who have recovered are protected from a second infection in the long‐term (e.g., ECDC, 2020; WHO, 2020).^3^ It therefore appears important that preventive behaviors are maintained post‐recovery, at least until evidence regarding long‐lasting COVID‐19 immunity is conclusive, or until the current vaccines have been widely administered. The concept of immunity passports—and incautious information like the above tweet—however, are likely to influence public perceptions of infection‐acquired COVID‐19 immunity and the appropriateness of going back to normal behavior post‐recovery. The current research investigates these perceptions, and in particular whether exposure to the idea of immunity passports and incautious information negatively affect COVID‐19 behavioral intentions post‐recovery. We also consider ways to lessen these negative effects.

### 1.1 COVID‐19 immunity and behavior

Early on during the pandemic, many optimistic headlines gave the impression that it is unlikely for people to be infected with COVID‐19 more than once (e.g., Mullin, 2020; Randall, 2020). In contrast, global health organizations have reported that it is too early to conclude whether or not recovery from COVID‐19 will equate to long‐lasting immunity. For example, the World Health Organisation has cautioned that there is currently no evidence that recovery from COVID‐19 confers protective immunity to repeated infection (WHO, 2020). A report by the European Centre for Disease Prevention and Control cautioned that protective immunity from COVID‐19 may only last around six months (ECDC, 2020). The UK’s Scientific Advisory Group for Emergencies have further cautioned that indirect transmission of COVID‐19 is still possible (SAGE, 2020a), and advised that knowledge of a person’s immunity status cannot be relied upon to enable a change in behavior, without risking serious implications (SAGE, 2020b).

Conflicting information is likely to affect the extent to which people are willing to take preventive action against COVID‐19. For instance, although health reports state that the science on infection‐acquired COVID‐19 immunity is inconclusive, governments hinting at the possibility of issuing immunity passports to people who have recovered (McMillan, 2020) may give the opposite impression. This could be considered an *unintended effect* of health communication (see Cho & Salmon, 2007 for a review). That is, where the intention may have been to inform the public of possible measures to ease COVID‐19 restrictions, the unintended effect is that it may trigger the assumption that recovery equates to immunity, leading to lower engagement with preventive behaviors in people who have recovered.

Unintended effects have been highlighted by previous research on health messaging. For example, an AIDS prevention campaign promoting the message “talk to your partner” was associated with increased sexual activity without increasing the use of condoms (Welch Cline et al., 1992). Other research has shown that fear appeal messaging (i.e., highlighting negative consequences) aimed at promoting preventative behaviors against skin cancer also had counterintuitive effects, specifically for people in the earlier decision‐making stages of taking on preventive action (Cho & Salmon, 2006). Among other reasons, Cho and Salmon (2007) argued that these effects can occur as a result of confusion or misunderstanding (i.e., obfuscation), a boomerang effect due to psychological avoidance, and a decrease in perceived risk as a result of message exposure. Therefore, considering the potential unintended effects of health messaging on COVID‐19 immunity is important.

Indeed, SAGE have highlighted that antibody testing may negatively influence such COVID‐19 preventative behaviors (SAGE, 2020c). Supporting these concerns, Smith and colleagues (2020) found that people who *suspect* they had COVID‐19 in the past were more likely to agree that they have some immunity, reported lower social distancing intentions, and were less worried about COVID‐19, compared to people who did not suspect so. Furthermore, Waller and colleagues (2020) found that people given a hypothetical *positive* antibody test result, named “immunity” perceived lower risk of reinfection, believed that they had some immunity, and reported lower social distancing and hygiene intentions, compared to people who were given the same test result named “antibody”. These emerging results suggest that appropriate communication about infection‐acquired COVID‐19 immunity will be crucial for as long as preventive behaviors are necessary. It is this matter that we turn to in the current research.

### 1.2 The present research

In two studies, we examined the effects of perceived recovery, and information about immunity passports, on intentions to mitigate the spread of COVID‐19. In Study 1, we examined the effects of *imagining* recovery from COVID‐19 on social distancing and hygiene intentions, compared to a control group. We also examined the extent to which imagining being currently infected influences these factors. Finally, we compared behavioral intentions and worry about COVID‐19 between people who *suspect that* they were infected in the past and people who did not suspect so, and whether certainty of suspected COVID‐19 status moderates these relationships (i.e., people who are more certain of their past infection may have lower behavioral intentions compared to people who are less certain). In Study 2, we focused on the effects of being exposed to the concept of immunity passports, and incautious versus cautious information regarding immunity, on social distancing, hygiene, and face‐covering intentions. Again, we also examined the effect of suspected COVID‐19 status, with certainty of said status as a potential moderator.^4^

In both studies, we recruited participants by means of convenience sampling. Study 1 was advertised on three social media platforms: Facebook, Twitter, and Reddit (the majority of participants were recruited from the latter), and Study 2 was solely advertised on Reddit. In all cases, the same advertisement was posted on each platform, referring to a survey on COVID‐19 that was being conducted by psychology researchers at the University of Kent. No incentives were offered. Our efforts were mainly focused on Reddit for several reasons: (1) Reddit now boasts 52 million daily active users worldwide (Reddit, 2020), with just over half of its traffic coming from the US (SimilarWeb, 2020), followed by the UK and Canada at 8% each; (2) scholars have documented the validity of using Reddit to obtain large inexpensive samples, including research on public health messaging (see Record et al., 2018), and findings from participants recruited via Reddit replicate previous findings in the psychological literature (e.g., Biddlestone et al., 2020; Jamnik & Lane, 2017); and (3) numerous forum pages (known as “subreddits”) dedicated specifically to information about COVID‐19 were created early on in the pandemic (e.g., r/TexasCoronavirus; r/CoronavirusUK). Our recruitment strategy therefore afforded us the opportunity to distribute our survey widely and obtain high‐powered samples. Both studies were designed and administered using the Qualtrics questionnaire design software.^5^ Hypotheses, analyses, and materials for both studies were pre‐registered, and the documentation can be found here: https://www.osf.io/6qtmy/registrations

## 2 STUDY 1

In Study 1, we predicted that people who *imagined* they had *recovered* from COVID‐19 would show lower COVID‐19 preventive intentions (e.g., Waller et al., 2020), compared to a control group. We also expected that participants who *imagined* that they were *currently infected* would show higher intentions compared to a control group. This prediction was inspired by news reports (e.g., Birch, 2020; McDonnell, 2020) advising people to “act like you have it [COVID‐19]” since asymptomatic people may be “super‐spreaders” (Li et al., 2020), which at the time of writing was the UK government’s mainline message to the public (BBC, 2021).

Finally, we predicted participants who *suspect* that they were infected in the past to show lower COVID‐19 preventive intentions, and less worry, than the control group (Smith et al., 2020), at higher levels of certainty of past infection only. To establish this comparison group, we asked questions at the beginning of the study (and Study 2) to ascertain participants’ COVID‐19 status. This process placed participants into the following groups; *a*) not tested and do not suspect to be currently infected; *b*) not tested and do not suspect to be currently infected, but suspect to have had it in the past; *c*) not tested, but suspect to be currently infected, *d*) have been tested and results were negative; *e*) have been tested, results were positive and they were still infected; *f*) have been tested, results were positive but they had since recovered; and *g*) have been tested, results were positive but they were unsure of their recovery status. All participants completed the dependent measures, but in Study 1, only group *a* were assigned to the experimental conditions, and group *b* was used as a comparison group for the moderation analyses.

### 2.1 Methods

#### 2.1.1 Participants and design

G*Power determined that in order to detect a small effect (*d* = 0.20) with a power of 0.80, at least 394 participants were required in each experimental group. We recruited 1768 participants from 13th to 14th May 2020 via posts on social media (i.e., Facebook and Twitter) and Reddit forums. Vaccines against COVID‐19 were not available at this time. Participants who suspected that they do not currently have COVID‐19 (group *a*: *n* = 1,205) and those who suspected they had it in the past (group *b*: *n* = 426) were included in the analyses, and the remaining participants were excluded (*n* = 137).^6^ We also excluded participants who failed at least one of the two attention checks (*n* = 28). The remaining participants (*N* = 1604; 881 women, 654 men, 24 trans, 32 rather not say, *M_age_* = 34.09 years, *SD* = 10.4, range = 18–78 years) were included in the final analyses. Of these participants, 65.1% were American, 22.4% were UK nationals, and the remaining 12.5% were made up of 58 different nationalities; 4% were frontline healthcare workers and the remaining 96% were not; finally, 30% had an underlying health condition, 57% did not, and the remaining 13% were not sure.^7^

The first part of the study was experimental, including three conditions (“recovered”, “infected”, and control), consisting of participants from group *a* only. Social distancing and hygiene intentions were the dependent variables. The second part of the study was correlational, examining whether any differences in intentions and worry about COVID‐19 between the control (from group *a*) and group *b*, are moderated by certainty of suspected COVID‐19 status.

#### 2.1.2 Materials and procedure

Participants were asked questions about their current COVID‐19 status. They were asked if they have been tested for COVID‐19 (*yes* or *no*). Participants who reported *yes* were then asked what the result of their test was (*positive* or *negative*). Participants who reported *positive* were then asked about their current condition (*still have it*, *recovered*, or *not sure*). Participants who reported *no* to being tested were then asked whether they suspect they currently had COVID‐19 (*yes*, *no*, or *no, but I suspect I had it in the past*), and how certain they are of this (1 = *I am not confident*, 5 = *I am confident*).

Participants from group *a* were then randomly assigned to one of three conditions: “recovered”, “infected”, and control. Participants in the “recovered” condition were asked to “*…imagine that you had a* *Coronavirus* *test in the past and that your results were positive… However, you are now fully recovered*”. Participants in the “infected” condition were asked to “*…imagine that you have had a* *Coronavirus* *test recently and your results are positive*”). In both conditions, participants were then asked to try to put themselves in the shoes of someone with their imagined COVID‐19 status and to think about how this would make them feel. Participants in the control condition were not presented with any information.

All participants were then asked to report how worried they were about COVID‐19 (one item, 1 = *Not at all worried*, 7 = *Extremely* *worried*).^8^ To maximize engagement with the manipulations, participants in the experimental conditions were additionally asked to report how vivid and clear (one item each) their thoughts were each on a 7‐point scale (1 = *not at all*, 7 = *very much*), and to describe them in a textbox.

All participants were then asked to report their social distancing (7 items; e.g., “Remain at least 2 meters (6 feet) apart from other people”; α = 0.87) and hygiene (3 items; e.g., “Wash your hands after every outing”; α = 0.62) intentions over the next month (1 = *definitely not*, 5 = *definitely yes*; adapted from Biddlestone et al., 2020). Participants in the experimental conditions were asked to keep their imagined COVID‐19 status in mind when answering these questions.

Finally, all participants completed several measures that were included as covariates; age, gender, education (1 = *no formal education*, 2 = *elementary level*, 3 = *middle school level*, 4 = *high school level*, 5 = *college or university [Bachelor’s* *degree],* 6 = *college or university [Graduate degree]*, whether they have any relevant underlying health conditions (*yes*, *no*, or *not sure*), and whether they work in frontline healthcare (*yes* or *no*), before being debriefed and thanked.

### 2.2 Results

See Table 1 for means, standard deviations, standard errors for both social distancing and hygiene intentions, and worry about COVID‐19 by condition/group.

**TABLE 1:** Means, standard deviations, and standard error of mean for social distancing, hygiene, and face covering intentions over the next month, and feelings of worry about COVID−19, by conditon, for Studies 1 and 2.

Study #	Condition or group	Size	Social distancing			Hygiene			Face covering			COVID‐19 worry		
			*M*	*SD*	SE	*M*	*SD*	SE	*M*	*SD*	SE	*M*	*SD*	SE
Study 1	Control	*n* = 417	4.21	0.78	0.04	4.63	0.56	0.03	–	–	–	4.85	1.47	0.07
	“Recovered”	*n* = 381	4.09	0.91	0.05	4.67	0.53	0.03	–	–	–	–	–	–
	“Infected”	*n* = 387	4.81	0.45	0.02	4.78	0.45	0.02	–	–	–	–	–	–
	Group *b*	*n* = 419	4.15	0.83	0.04	4.64	0.54	0.03	–	–	–	4.70	1.43	0.07
Study 2	Control	*n* = 480	3.98	0.85	0.04	4.59	0.57	0.03	4.40	0.93	0.04	–	–	–
	“Immunity”	*n* = 403	3.70	0.96	0.05	4.59	0.56	0.03	4.08	1.16	0.06	–	–	–
	“Incautious”	*n* = 430	3.61	0.97	0.05	4.62	0.58	0.03	4.10	1.17	0.06	–	–	–
	“Cautious”	*n* = 420	4.01	0.86	0.04	4.65	0.56	0.03	4.39	0.98	0.05	–	–	–
	Group *a*	*n* = 374	4.00	0.88	0.05	4.59	0.57	0.03	4.39	0.96	0.05	4.52	1.55	0.08
	Group *b*	*n* = 106	3.92	0.74	0.07	4.57	0.59	0.06	4.42	0.81	0.08	4.56	1.39	0.14

Note: For Study 1, all experimental conditions, including the control, consist of participants from group *a* (not tested and do not suspect to be currently infected) only. For Study 2, all experimental conditions, including the control, consist of participants from groups *a* and *b* (not tested and do not suspect to be currently infected, but suspect to have had it in the past) only. Furthermore, for Study 2, groups *a* and *b* consist of participants from the control group only.

#### 2.2.1 Imagined COVID‐19 status

Two regression models tested whether “recovered” significantly decreased, and “infected” significantly increased, social distancing and hygiene intentions, compared to the control. We created two dummy coded variables representing the following contrasts: X1 = (0) control versus (1) “infected”; X2 = (0) control versus (1) “recovered”. Social distancing and hygiene intentions were entered as dependent variables. All covariate measures were included.

The regression models were significant and accounted for 18% and 5% of the variance in social distancing and hygiene intentions respectively (Table 2). Compared to the control condition, “recovered” significantly decreased (Cohen’s *d* = 0.14), and “infected” significantly increased (Cohen’s *d* = 0.93), social distancing intentions. Only “infected” significantly increased (Cohen’s *d* = 0.29) hygiene intentions. Of the covariates, age, gender (*female*), and underlying health conditions (*yes*) positively predicted all outcomes.

**TABLE 2:** Predictors of social distancing and hygiene intentions over the next month, for the experimental conditions (Study 1)

Variable	Social distancing				Hygiene			
	*B*	95% CI	*β*	*p*	*B*	95% CI	*β*	*p*
1. “Recovered” versus control	−0.14	[−0.25, −0.03]	−0.08	.015	0.03	[−0.04, 0.11]	0.03	.401
2. “Infected” versus control	0.60	[0.49, 0.72]	0.34	<.001	0.17	[0.09, 0.25]	0.15	<.001
3. Age	0.01	[0.01, 0.01]	0.07	.032	0.01	[0.01, 0.01]	0.08	.011
4. Gender (Male = 0, Female = 1)	0.16	[0.07, 0.26]	0.10	.001	0.13	[0.07, 0.20]	0.13	<.001
5. Underlying health condition (No = 0, Yes = 1)	0.15	[0.05, 0.25]	0.09	.004	0.08	[0.01, 0.15]	0.07	.028
6. Frontline healthcare (No = 0, Yes = 1)	0.22	[−0.03, 0.47]	0.05	.089	−0.04	[−0.21, 0.13]	−0.02	.613
7. Education	0.04	[−0.02, 0.09]	0.04	.230	0.02	[−0.02, 0.06]	0.04	.269
*R* ^2^	0.17				0.05			
*F*	*F*(7, 972) = 30.53*				*F*(7, 972) = 7.92*			

Note

Control = 0; “recovered” and “infected” = 1.

* *p* < .001.

#### 2.2.2 Suspected COVID‐19 status

To test whether *suspected COVID‐19 status* (group *a* versus group *b*) predicted worry about COVID‐19, social distancing, and hygiene intentions, and whether these relationships were moderated by *certainty of suspected COVID‐19 status*, we performed a series of hierarchal regression analyses. We created a dummy coded variable representing suspected COVID‐19 status: 0 = group *a* versus 1 = group *b*. For all dependent variables, Step 1 tested the main effects of suspected COVID‐19 status (predictor) and certainty of said status (moderator) and also included all covariates. In Step 2, we added the two‐way interaction of the predictor and moderator. The continuous moderator variable was mean‐centered prior to analyses.

In Step 1, suspected COVID‐19 status did not predict social distancing (*β* = −0.07, *p* = .080; *R* ^2^ = 0.07, *F*(7, 688) = 7.48, *p* < .001), hygiene intentions (*β* = −0.01, *p* = .970; *R* ^2^ = 0.04, *F*(7, 688) = 4.41, *p* < .001), or worry about COVID‐19 (*β* = −0.07, *p* = .094; *R* ^2^ = 0.13, *F*(7, 688) = 15.00, *p* < .001). Certainty of suspected COVID‐19 status negatively predicted worry about COVID‐19 (*β* = −0.10, *p* = .008), but not social distancing (*β* = −0.08, *p* = .061) or hygiene intentions (*β* = 0.03, *p* = .504). In Step 2, the interaction term did not significantly increase the variance for social distancing (*β* = −0.01, *p* = .888; *ΔR* ^2^ = 0.01, *ΔF*(1, 687) = 0.02, *p* = .888), hygiene intentions (*β* = 0.02, *p* = .787; *ΔR* ^2^ = 0.01, *ΔF*(1, 687) = 0.07, *p* = .787), or worry about COVID‐19 (*β* = 0.09, *p* = .093; *ΔR* ^2^ = 0.01, *ΔF*(1, 687) = 2.83, *p* = .093). Thus, we did not find the expected moderation effects.

## 3 STUDY 2

Study 1 demonstrated that *imagining* recovery from COVID‐19 decreased social distancing intentions, and imagining being infected increased hygiene and social distancing intentions, relative to a control group. However, *suspected* COVID‐19 status did not predict intentions or worry about COVID‐19, even when including certainty of COVID‐19 status as a moderator. In Study 2, we sought to extend the effect of imagined recovery by explicitly mentioning the concept of immunity passports. Specifically, instead of comparing the effect of antibody test result framing on behavioral intentions (Waller et al., 2020), we examined how—compared to a control group—exposure to more or less cautious information regarding infection‐acquired COVID‐19 immunity and immunity passports may influence preventive behaviors. We also included face covering intentions as a new DV. Since in Study 1 we found no difference in behavioral intentions between people who suspect they had COVID‐19 in the past compared with people who did not suspect so (contrary to Smith et al., 2020), we included both of these groups (*a* and *b*) in the experimental conditions. However, by splitting the control condition in the current study, this still allowed us to compare group *a* and *b’s* COVID‐19 preventive intentions and worry about COVID‐19, and whether these relationships are moderated by certainty of suspected COVID‐19 status.

All participants in the experimental conditions were asked to imagine that they had recovered from COVID‐19 and were then exposed to the concept of immunity passports. These participants were then split randomly into three conditions where they received: (1) no further information, (2) incautious information about infection‐acquired COVID‐19 immunity, or (3) cautious information about infection‐acquired COVID‐19 immunity. Finally, there was also a control group, wherein participants were not asked to imagine past infection and were not presented with any information about immunity or immunity passports. Relative to the control group, we expected (1) and (2) to indicate incremental reduced behavioral intentions, but that (3) would display similar levels of behavioral intentions.

### 3.1 Methods

#### 3.1.1 Participants and design

As in Study 1, G*Power determined that in order to detect a small effect (*d* = 0.20) with a power of 0.80, at least 394 participants were required in each experimental group. We recruited 1999 participants from 8th to 12th June 2020 via posts on Reddit forums. Vaccines against COVID‐19 were not available at this time. Participants who suspected that they did not currently have COVID‐19 (group *a*: *n* = 1,370) and those who suspected they had it in the past (group *b*: *n* = 387) remained and all other participants were excluded (*n* = 242).^9^ We also excluded participants who failed at least one of two attention checks (*n* = 24). The remaining participants (*N* = 1733; 846 men, 826 women, 20 trans, 32 rather not say, M_age_ = 33.62 years, *SD* = 10.80, range = 18–73 years) were included in the final analyses. Of these participants, 56.6% were American, 27.7% were UK nationals, and the remaining 15.7% were made up of 50 different nationalities; 4% were frontline healthcare workers and the remaining 96% were not; finally, 25% had an underlying health condition, 62% did not, and the remaining 13% were not sure.^10^

As in Study 1, the first part of the study was experimental, including four conditions (“immunity”, “incautious”, “cautious”, and control), consisting of participants from groups *a* and *b*. Social distancing, face covering and hygiene intentions were the dependent variables. The second part of the study was correlational, examining whether any differences between group *a* and group *b* (consisting of participants from the control only) in behavioral intentions, and worry about COVID‐19, are moderated by certainty of suspected COVID‐19 status.

#### 3.1.2 Materials and procedure

Participants were asked the same series of questions about their current COVID‐19 status as in Study 1, including certainty of suspected status.

Participants from groups *a* and *b* were then randomly assigned to one of four conditions; “immunity”, “incautious [information about immunity]”, “cautious [information about immunity]”, and a control group. As in Study 1, participants in the “immunity”, “incautious”, and “cautious” conditions were asked to imagine that they have recovered from COVID‐19. Additionally, they were then presented with brief information regarding the concept of “immunity passports” (i.e., *Some governments have suggested that the detection of antibodies in people who have recovered from* *Coronavirus* *could serve as the basis for an “immunity passport” … that would enable individuals to travel or to return to work*). Participants in the “incautious” condition were further presented with two incautious, and participants in the “cautious” condition were presented with two cautious, statements regarding evidence of immunity (i.e., “…*early evidence [suggests]/[does not conclude] that antibodies [equals]/[equal] immunity, and that subsequent infection [is not likely]/[remains possible]*” and “*It is [unlikely]/[still likely] that individuals who have recovered from* *Coronavirus* *will directly or indirectly spread the virus onto others*”). In all three experimental conditions, participants were asked to keep their imagined COVID‐19 status in mind and to let the additional information about immunity sink in. Participants in the control condition were not presented with any information about immunity passports and were not asked to imagine that they had been infected in the past. As in Study 1, all participants were then asked to report how worried they are about COVID‐19 and participants in the experimental conditions only were also asked to report how vivid and clear their thoughts were, and to describe them in a textbox.

All participants then reported their social distancing (α = 0.88) and hygiene (α = 0.63) intentions, as in Study 1. Two new items measured face covering intentions (e.g., “Wear a face make in busy social situations”; *r* = 0.79). As in Study 1, participants in the three experimental conditions were asked to keep their imagined COVID‐19 status in mind when answering these questions. Finally, all participants completed the covariate measures, and were then debriefed and thanked, as in Study 1.

### 3.2 Results

See Table 1 for means, standard deviations, and standard errors for social distancing, hygiene, and face covering intentions, and worry about COVID‐19, by condition/group.

#### 3.2.1 Imagined COVID‐19 status

Three regression models tested whether the concept of “immunity” and “incautious” information significantly decreased, and “cautious” information did not increase or decrease, social distancing, hygiene, and face covering intentions, compared to the control condition. We created three dummy coded variables representing the following contrasts: X1 = (0) “immunity” versus (1) control; X2 = (0) “incautious” versus (1) control, X3 = (0) “cautious” versus (1) control. Social distancing, hygiene, and face covering intentions were entered as dependent variables.

All regression models accounted for significant variance (Table 3). Compared to the control, “immunity” and “incautious” information significantly decreased (Cohen’s *d* = 0.31, Cohen’s *d* = 0.41, respectively), and “cautious” information had no effect on, social distancing intentions. Only “cautious” information significantly increased (Cohen’s *d* = 0.11) hygiene intentions. “Immunity” and “incautious” information significantly decreased (Cohen’s *d* = 0.31, Cohen’s *d* = 0.29, respectively), and “cautious” information had no effect on, face covering intentions. Of the covariates, age, gender (*female*), and underlying health condition (*yes*) positively predicted all outcomes.

**TABLE 3:** Predictors of social distancing, hygiene, and face covering intentions over the next month, for the experimental conditions (Study 2)

Variable	Social distancing				Hygiene				Face covering			
	*B*	95% CI	*β*	*p*	*B*	95% CI	*β*	*p*	*B*	95% CI	*β*	*p*
1. “Immunity” versus control	−0.31	[−0.44, −0.18]	−0.14	<.001	−0.01	[−0.08, 0.08]	−0.01	0.922	−0.36	[−0.51, −0.20]	−0.14	<.001
2. “Incautious” versus control	−0.38	[−0.51, −0.26]	−0.18	<.001	0.03	[−0.05, 0.11]	0.03	0.417	−0.29	[−0.44, −0.15]	−0.12	<.001
3. “Cautious” versus control	0.02	[−0.11, 0.15]	0.01	.801	0.08	[0.01, 0.16]	0.06	0.044	−0.03	[−0.18, 0.12]	−0.01	.692
4. Age	0.01	[0.01, 0.01]	0.07	.005	0.01	[0.01, 0.01]	0.06	0.029	0.01	[0.01, 0.01]	0.07	.008
5. Gender (Male = 0, Female = 1)	0.28	[0.19, 0.38]	0.15	<.001	0.17	[0.12, 0.23]	0.16	< 0.001	0.43	[0.32, 0.54]	0.20	<.001
6. Underlying health condition (No = 0, Yes = 1)	0.30	[0.19, 0.40]	0.14	<.001	0.08	[0.02, 0.15]	0.07	0.013	0.27	[0.15, 0.40]	0.11	<.001
7. Frontline healthcare (No = 0, Yes = 1)	−0.07	[−0.30, 0.17]	−0.01	.586	−0.04	[−0.18, 0.11]	−0.01	0.602	−0.11	[−0.39, 0.16]	−0.02	.412
8. Education	−0.01	[−0.07, 0.04]	−0.01	.624	−0.02	[−0.06, 0.01]	−0.03	0.250	0.01	[−0.07, 0.07]	0.01	.988
*R* ^2^	0.09				0.04				0.09			
*F*	*F*(8, 1,453) = 18.87*				*F*(8, 1,453) = 7.63*				*F*(8, 1,453) = 16.91*			

Note

Control = 0; “immunity”, “incautious”, and “cautious” = 1.

* *p* <.001.

#### 3.2.2 Suspected COVID‐19 status

As in Study 1, we performed analyses testing whether *certainty of suspected COVID‐19 status* moderated the effect of *suspected COVID‐19 status* (0 = group *a* versus 1 = group *b*) on social distancing, hygiene and face covering intentions, and worry about COVID‐19.

In Step 1, suspected COVID‐19 status did not predict social distancing (*β* = −0.06, *p* = .274; *R* ^2^ = 0.11, *F*(7, 400) = 6.92, *p* < .001), hygiene (*β* = 0.01, *p* = .829; *R* ^2^ = 0.02, *F*(7, 400) = 1.15, *p* = .331), face covering intentions (*β* = −0.02, *p* = .773; *R* ^2^ = 0.10, *F*(7, 400) = 6.01, *p* < .001), or worry about COVID‐19 (*β* = −0.01, *p* = .976; *R* ^2^ = 0.08, *F*(7, 400) = 4.61, *p* < .001). Certainty of suspected COVID‐19 status negatively predicted social distancing (*β* = −0.11, *p* = .039), face covering intentions (*β* = −0.16, *p* = .002), and worry about COVID‐19 (*β* = −0.14, *p* = .008), but not hygiene intentions (*β* = 0.03, *p* = .547).

**FIGURE 1:**
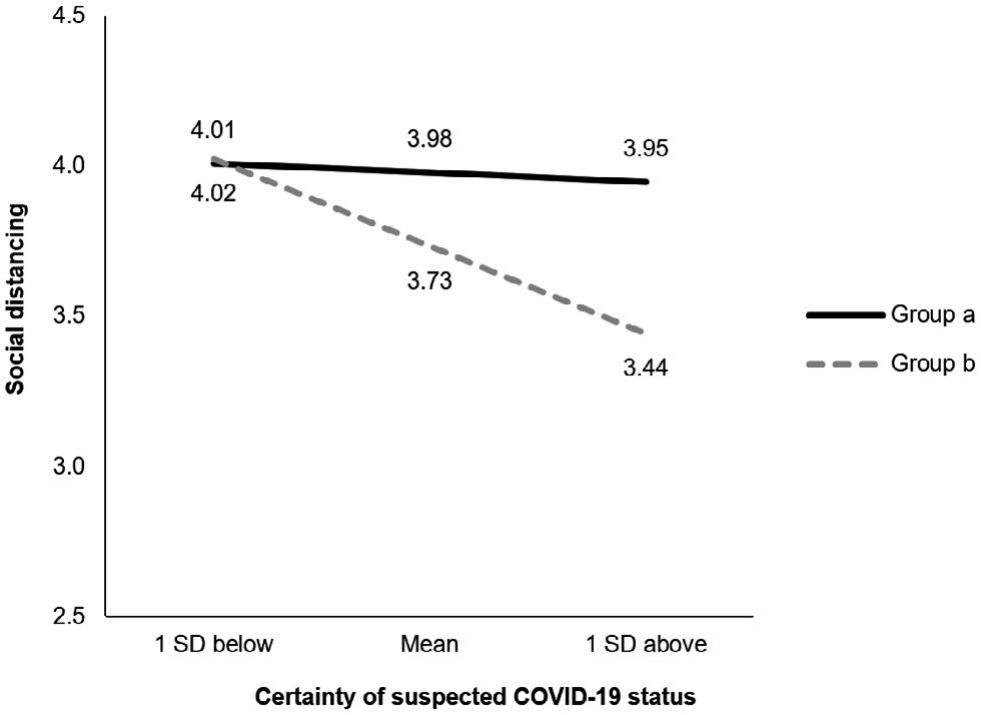
Moderating effect of certainty of suspected COVID‐19 status on social distancing intentions. At low certainty, there is no difference in social distancing intentions between either suspected COVID‐19 status groups (95% CI [−0.25, 0.22]). However, as certainty increases to mid (95% CI [0.01, 0.48]) and high levels (95% CI [0.13, 0.87]), suspected recovery from COVID‐19 (group *b*) reduces social distancing intentions, compared to people who do not suspect they were infected in the past (group *a*)

In Step 2, the interaction term significantly accounted for increased variance for social distancing (*β* = −0.16, *p* = .013; *ΔR* ^2^ = .01, *ΔF*(1, 399) = 6.22, *p* = .013), but not for hygiene (*β* = −0.05, *p* = .418; *ΔR* ^2^ = 0.01, *ΔF*(1, 399) = 0.66, *p* = .418), face covering intentions (*β* = −0.02, *p* = .816; *ΔR* ^2^ = 0.01, *ΔF*(1, 399) = 0.05, *p* = .816), or worry about COVID‐19 (*β* = −0.04, *p* = .531; *ΔR* ^2^ = 0.01, *ΔF*(1, 399) = 0.39, *p* = .531). Simple slopes analysis using PROCESS (Model 1, Hayes, 2013) revealed that the relationship between certainty of suspected COVID‐19 status and social distancing intentions was negative and significant for group *b* (*β* = −0.36, *p* < .001) but was not significant for group *a* (*β* = −0.04, *p* = .502; Figure 1).

## 4 GENERAL DISCUSSION

In the current research, imagined past infection from COVID‐19 reduced participants’ COVID‐19 preventive intentions compared to a control group. In Study 1, when participants imagined that they had recovered from COVID‐19, they reported lower social distancing intentions than the control group, although there were no differences in hygiene intentions. This finding was replicated in Study 2, where participants were also presented with the concept of an immunity passport. Here, we also found a negative effect on face covering intentions, a negative effect on social distancing intentions, but no differences in hygiene intentions, as in Study 1.

Crucially, however, we showed that these effects were worsened when participants were presented with incautious information about infection‐acquired COVID‐19 immunity (again, for social distancing intentions only), but were attenuated when cautious information was presented (for both social distancing and face covering intentions). Furthermore, hygiene intentions were improved compared to the control group when cautious information was presented about immunity. These findings partially support recent research demonstrating that perceived infection‐acquired immunity may be damaging to social distancing and hygiene intentions (Waller et al., 2020), and supplements existing research on the unintended effects of health communication (Cho & Salmon, 2007). However, we did not find support for a decrease in hygiene practices. Furthermore, we extend these recent findings by showing that the concept of immunity passports may be dealt with more effectively if information is presented with sensitivity toward the current scientific consensus concerning infection‐acquired COVID‐19 immunity.

We also investigated whether people who *suspect that* they had COVID‐19 in the past would have lower COVID‐19 preventive intentions and worry less about COVID‐19 than people who do not suspect so. Contrary with previous findings (Smith et al., 2020), suspected COVID‐19 status did not predict intentions or worry about COVID‐19 in either study. However, in Study 2, higher levels of certainty of past infection moderated the effect of social distancing intentions, which were significantly lower compared to the control group. Taking the experimental and comparison analyses together, the results suggest that when people are highly certain of past infection, through suspicion or testing, then social distancing and face covering intentions are likely to decrease. However, there were no such decreases for hygiene intentions, in any setting.

Finally, in Study 1 we showed when participants imagined that they were currently infected with COVID‐19 this increased their social distancing and hygiene intentions compared to the control group. These findings echo the sentiments of many news articles and government initiatives encouraging people to “act like you have it” (e.g., BBC, 2021; Birch, 2020). Particular focus on imagined current infection is therefore a promising behavioral intervention strategy.

### 4.1 Limitations and future research

Despite the conceptual and methodological advancements of the current paper, some limitations could be addressed in future investigations. Firstly, the current research did not account for the potential influence of symptom severity on behavioral intentions. For example, if participants were asked to imagine that they have recovered from COVID‐19 and they were asymptomatic, they may demonstrate lower intentions than participants who were asked to imagine they suffered with more severe symptoms. Secondly, we did not control for the source of information presented. Research suggests that people are more likely to perceive legitimate leadership when they identify strongly with the leader (e.g., Hogg et al., 2005). Therefore, it may be the case that stronger identification with leaders that speak incautiously or cautiously about COVID‐19 immunity further exacerbates or attenuates (respectively) the negative effects of imagined recovery on intentions.

Thirdly, although *actual* COVID‐19 status was measured in the current findings, the sample sizes allowing us to analyze these effects were comparably smaller than the sample sizes for the *suspected* COVID‐19 status groups (*a* and *b*). Thus, the literature would benefit from replicating the current findings in designs that focus on people who have actually recovered from COVID‐19. Fourth, despite the large sample sizes, participant recruitment relied heavily on Reddit as its main source. Certain demographics (i.e., mean age) collected in the current studies were comparable to those in research that uses popular crowdsourcing platforms (e.g., Amazon’s Mechanical Turk, Green & Douglas, 2018; Prolific Academic, M. Biddlestone, A. Cichocka, M. Główczewski, A. Cislak, A, under review). However, it has been noted that the majority of Reddit users appear to be white males (Barthel et al., 2016). Therefore, although the current research conceptually replicated recent findings (e.g., Smith et al., 2020; Waller et al., 2020), future research would benefit from replications on nationally representative samples, additionally controlling for a wider range of demographic variables (e.g., ethnicity). Finally, this paper is the first to experimentally demonstrate the potential positive effects of “acting like you have it [COVID‐19]” on preventive behaviors, but does not indicate when and how this intervention might be best applied in the real world. Taking this idea further is a promising approach to improving people’s COVID‐19 behavioral intentions.

## 5 CONCLUSION

Governments have suggested introducing immunity passports (McMillan, 2020) despite the uncertainty surrounding infection‐acquired COVID‐19 immunity (e.g., ECDC, 2020; WHO, 2020). Although it is difficult to gauge the effects of these announcements directly, our findings suggest that premature public discussions based on inconclusive scientific research are likely to have had a negative effect on individuals’ willingness to comply with pandemic safety guidelines post‐recovery. Furthermore, our findings suggest that governments would benefit by prefacing or debriefing such announcements with cautious information regarding the uncertainty of COVID‐19 immunity, or perhaps to not announce these ideas until scientific certainty on the matter has been achieved.

## CONFLICTS OF INTEREST

The authors have declared no conflicts of interest for this article.

## Supporting information

Supplementary Material

## Data Availability

The data (including materials and pre‐registrations) that support the findings of these studies are openly available in Open Science Framework at https://www.osf.io/6qtmy/.

## Footnotes

^1^
Twitter promptly labeled this tweet as misleading, stating that it violated their rules about spreading misleading and potentially harmful information related to COVID‐19.

^2^
At the time of first submitting this manuscript, 38,317,724 people had recovered worldwide, according to https://www.worldometers.info/coronavirus/.

^3^
It is also still unknown how long vaccine‐acquired immunity will last (e.g., CDC, 2021).

^4^
The current studies were part of a wider project which also measured a range of other constructs such as attachment styles and belief in conspiracy theories.

^5^
For both studies, we checked the “prevent ballot stuffing” feature on Qualtrics to prevent the survey being taken multiple times by the same person.

^6^
These groups were not central to our hypotheses (this is the same for Study 2): *c*) *n* = 22; *d*) *n* = 110; *e*) *n* = 2; *f*) *n* = 2; and *g*) *n* = 3. Please see Supplementary Materials for further details.

^7^
The main results were unchanged when nationalities were controlled for and when all covariates were excluded. Please see Supplementary Materials for further details.

^8^
Participants in the experimental conditions were asked: “*How worried did this task make you feel about* *Coronavirus?”* The way in which we framed this question did not give us confidence that it measured worry about COVID‐19. Therefore, we only focused on worry when comparing the control group (from group *a*) against group *b*; this is the same for Study 2.

^9^

*c*) *n* = 12; *d*) *n* = 208; *e*) *n* = 7; *f*) *n* = 13; and *g*) *n* = 2. Please see Supplementary Materials for further details.

^10^
As in Study 1, the main results were unchanged when nationalities were controlled for and when all covariates were excluded. Please see Supplementary Materials for further details.

## References

[jasp12779-bib-0001] Barthel, M. , Stocking, G. , Holcomb, J. , & Mitchell, A. (2016, February 25). Reddit news users more likely to be male, young and digital in their news preferences. Pew Research Center. https://www.journalism.org/2016/02/25/reddit‐news‐users‐more‐likely‐to‐be‐male‐young‐and‐digital‐in‐their‐news‐preferences/

[jasp12779-bib-0002] BBC . (2021, January 9). COVID‐19: Act like you’ve got the virus, government urges. BBC News. https://www.BBC.co.uk/news/uk‐55598918

[jasp12779-bib-0004] Biddlestone, M. , Green, R. , & Douglas, K. M. (2020). Cultural orientation, power, belief in conspiracy theories, and intentions to reduce the spread of COVID‐19. British Journal of Social Psychology, 59(3), 663–673. 10.1111/bjso.12397 32592420 PMC7361833

[jasp12779-bib-0005] Birch, J. (2020, March 18). Act like you already have coronavirus. HuffPost. https://www.huffpost.com/entry/act‐like‐you‐have‐coronavirus_l_5e721aafc5b6eab7793fc120

[jasp12779-bib-0006] Centers for Disease Control and Prevention . (2021, January 25). Frequently asked questions about COVID‐19 vaccination. Retrieved February 3, 2021, from https://www.cdc.gov/coronavirus/2019‐ncov/vaccines/faq.html

[jasp12779-bib-0007] Cho, H. , & Salmon, C. T. (2006). Fear appeals for individuals in different stages of change: Intended and unintended effects and implications on public health campaigns. Health Communication, 20(1), 91–99. 10.1207/s15327027hc2001_9 16813492

[jasp12779-bib-0008] Cho, H. , & Salmon, C. T. (2007). Unintended effects of health communication campaigns. Journal of Communication, 57(2), 293–317. 10.1111/j.1460-2466.2007.00344.x

[jasp12779-bib-0009] European Centre for Disease Prevention and Control . (2020, June 30). Immune responses and immunity to SARS‐Cov‐2. Retrieved July 25, 2020, from https://www.ecdc.europa.eu/en/covid‐19/latest‐evidence/immune‐responses

[jasp12779-bib-0010] Green, R. , & Douglas, K. M. (2018). Anxious attachment and belief in conspiracy theories. Personality and Individual Differences, 125, 30–37. 10.1016/j.paid.2017.12.023

[jasp12779-bib-0028] Hayes, A. F. (2013). Methodology in the social sciences. Introduction to mediation, moderation, and conditional process analysis: A regression‐based approach. Guilford Press.

[jasp12779-bib-0011] Hogg, M. A. , Martin, R. , Epitropaki, O. , Mankad, A. , Svensson, A. , & Weeden, K. (2005). Effective leadership in salient groups: Revisiting leader‐member exchange theory from the perspective of the social identity theory of leadership. Personality and Social Psychology Bulletin, 31(7), 991–1004. 10.1177/0146167204273098 15951369

[jasp12779-bib-0012] Jamnik, M. R. , & Lane, D. J. (2017). The use of Reddit as an inexpensive source for high‐quality data. Practical Assessment, Research, and Evaluation, 22(5). 10.7275/swgt-rj52

[jasp12779-bib-0013] Li, R. , Pei, S. , Chen, B. , Song, Y. , Zhang, T. , Yang, W. , & Shaman, J. (2020). Substantial undocumented infection facilitates the rapid dissemination of novel coronavirus (SARS‐Cov‐2). Science, 368(6490), 489–493. 10.1126/science.abb3221 32179701 PMC7164387

[jasp12779-bib-0014] McDonnell, T. (2020, March 16). Behave like you have coronavirus. Quartz. https://www.qz.com/1819484/covid‐19‐is‐mostly‐spread‐by‐undetected‐people/

[jasp12779-bib-0015] McMillan, N. (2020, May 21). Immunity passports could help end lockdown, but risk class divides and intentional infections. The Conversation. https://www.theconversation.com/immunity‐passports‐could‐help‐end‐lockdown‐but‐risk‐class‐divides‐and‐intentional‐infections‐138513

[jasp12779-bib-0016] Mullin, G. (2020, March 13). BRACE YOURSELF: Millions in UK MUST catch coronavirus so we develop ‘herd immunity’, says top scientist.. The Sun. https://www.thesun.co.uk/news/11164977/millions‐uk‐coronavirus‐herd‐immunity/

[jasp12779-bib-0017] Randall, I. (2020, March 16). Monkeys CAN'T be reinfected with coronavirus in a sign 'herd immunity' WILL kick in as scientists dismiss fears patients could 'relapse' after recovery from the initial infection. MailOnline. https://www.dailymail.co.uk/sciencetech/article‐8116571/Monkeys‐reinfected‐coronavirus‐scientists‐find.html

[jasp12779-bib-0018] Record, R. A. , Silberman, W. R. , Santiago, J. E. , & Ham, T. (2018). I sought it, I Reddit: Examining health information engagement behaviors among Reddit users. Journal of Health Communication, 23(5), 470–476. 10.1080/10810730.2018.1465493 29718799

[jasp12779-bib-0019] Reddit . (2020, January). Reddit by numbers. Retrieved February 3, 2021, from https://www.redditinc.com/press

[jasp12779-bib-0020] Scientific Advisory Group for Emergencies (2020a). Transmission of SARS‐CoV‐2 and mitigating measures. GOV.UK. https://www.gov.uk/government/publications/transmission‐of‐sars‐cov‐2‐and‐mitigating‐measures‐update‐4‐june‐2020

[jasp12779-bib-0021] Scientific Advisory Group for Emergencies (2020b). Tests for antibodies against SARS‐Cov‐2. GOV.UK. https://www.gov.uk/government/publications/tests‐for‐antibodies‐against‐sars‐cov‐2‐2‐july‐2020

[jasp12779-bib-0022] Scientific Advisory Group for Emergencies (2020c). SPI‐B: Pre‐empting possible negative behavioral responses to COVID‐19 antibody testing. GOV.UK. https://www.gov.uk/government/publications/spi‐b‐pre‐empting‐possible‐negative‐behavioural‐responses‐to‐covid‐19‐antibody‐testing‐13‐april‐2020

[jasp12779-bib-0023] SimilarWeb . (2021, January). Reddit.com traffic statistics. Retrieved February 3, 2021, from https://www.similarweb.com/website/reddit.com/#overview

[jasp12779-bib-0024] Smith, L. E. , Mottershaw, A. L. , Egan, M. , Waller, J. , Marteau, T. M. , & Rubin, G. J. (2020). The impact of believing you have had COVID‐19 on self‐reported behavior: Cross‐sectional survey. PLoS One, 15(11), e0240399. 10.1371/journal.pone.0240399 33147219 PMC7641362

[jasp12779-bib-0025] Waller, J. , Rubin, G. J. , Potts, H. W. , Mottershaw, A. L. , & Marteau, T. M. (2020). ‘Immunity passports’ for SARS‐Cov‐2: An online experimental study of the impact of antibody test terminology on perceived risk and behavior. British Medical Journal Open, 10(8), e040448. 10.1136/bmjopen-2020-040448 PMC7462240 32868370

[jasp12779-bib-0026] Welch Cline, R. J. , Johnson, S. J. , & Freeman, K. E. (1992). Talk among sexual partners about AIDS: Interpersonal communication for risk reduction or risk enhancement? Health Communication, 4(1), 39–56. 10.1207/s15327027hc0401_4

[jasp12779-bib-0027] World Health Organization . (2020, April 24). “Immunity passports” in the context of COVID‐19. Retrieved July 25, 2020, from https://www.who.int/news‐room/commentaries/detail/immunity‐passports‐in‐the‐context‐of‐covid‐19

