# Supplementary material for: A call for caution regarding infection‐acquired COVID‐19 immunity: The potentially unintended effects of “immunity passports” and how to mitigate them
Source: J Appl Soc Psychol. 2021 May 29;51(7):720–9. doi: 10.1111/jasp.12779 (PMC8237004; doi:10.1111/jasp.12779)
Supplement: Supplementary file 1 — Supplementary Material [file JASP-51-720-s001.docx]

**Supplementary Materials**

**Controlling for nationality in the main analyses**

The results in the main text include all nationalities. Here we demonstrate that the pattern of results remains the same when nationalities are controlled for. In both studies, participants from the USA constituted the majority of the sample, United Kingdom was second, and the remainder of the sample was made up of 45-58 different nationalities. As participants from the USA took up over 50% of the sample in both studies, we dummy coded two contrasting nationality control variables: 0 = USA vs 1 = UK, and 0 = USA, 1 = Remaining. We then included these control variables into the main analyses. The main pattern of results did not change for Study 1 (see Table S1) or Study 2 (see Table S2, S3 and Figure S1).

| Table S1  ***Study 1****: Predictors of social distancing and hygiene intentions over the next month, for the experimental conditions (****controlling for nationality****).* | | | | | | | | |
| --- | --- | --- | --- | --- | --- | --- | --- | --- |
|  | Social distancing | | | | Hygiene | | | |
| Variable | *B* | 95% CI | β | *p* | *B* | 95% CI | β | *p* |
| 1. Recovered vs. control | -0.14 | [-0.25, -0.03] | -.08 | .015 | 0.04 | [-0.04, 0.11] | .03 | .343 |
| 1. Infected vs. control | 0.60 | [0.49, 0.72] | .34 | < .001 | 0.17 | [0.09, 0.25] | .15 | < .001 |
| 1. Age | 0.01 | [0.01, 0.01] | .07 | .032 | 0.01 | [0.01, 0.01] | .08 | .016 |
| 1. Gender (Male = 0, Female = 1) | 0.16 | [0.07, 0.26] | .10 | .001 | 0.12 | [0.06, 0.19] | .12 | < .001 |
| 1. Underlying health condition (No = 0, Yes = 1) | 0.15 | [0.05, 0.25] | .09 | .005 | 0.06 | [-0.01, 0.13] | .06 | .076 |
| 1. Frontline healthcare (No = 0, Yes = 1) | 0.21 | [-0.04, 0.46] | .05 | .094 | -0.06 | [-0.23, 0.11] | -.02 | .458 |
| 1. Education | 0.03 | [-0.02, 0.09] | .03 | .244 | 0.02 | [-0.02, 0.06] | .03 | .307 |
| 1. USA = 0 vs UK = 1 | -0.02 | [-0.13, 0.10] | -.01 | .779 | -0.19 | [-0.26, -0.11] | -.16 | < .001 |
| 1. USA = 0 vs Remaining = 1 | 0.02 | [-0.13, 0.18] | .01 | .757 | -0.03 | [-0.13, 0.07] | -.02 | .548 |
| *R*^2^ | .17 | | | | .07 | | | |
| *F* | *F*(9, 970) = 23.728* | | | | *F*(9, 970) = 8.954* | | | |
| ⁎ *p* < .001. | | | | | | | | |

| Table S2  ***Study* 2**: *Predictors of social distancing, hygiene, and face covering intentions over the next month, for the experimental conditions (****controlling for nationality****).* | | | | | | | | | | | | |
| --- | --- | --- | --- | --- | --- | --- | --- | --- | --- | --- | --- | --- |
|  | Social distancing | | | | Hygiene | | | | Face covering | | | |
| Variable | *B* | 95% CI | β | *p* | *B* | 95% CI | β | *p* | *B* | 95% CI | β | *p* |
| 1. Immunity vs. control | -0.30 | [-0.43, -0.17] | -.04 | < .001 | -0.01 | [-0.09, 0.07] | -.01 | .802 | -0.37 | [-0.51, -0.20] | -.14 | < .001 |
| 1. Incautious vs. control | -0.38 | [-0.50, -0.25] | -.18 | < .001 | 0.03 | [-0.05, 0.11] | .02 | .465 | -0.30 | [-0.44, -0.15] | -.12 | < .001 |
| 1. Cautious vs. control | 0.02 | [-0.11, 0.15] | .01 | .732 | 0.08 | [-0.01, 0.16] | .06 | .058 | -0.04 | [-0.18, 0.12] | -.01 | .637 |
| 1. Age | 0.01 | [0.01, 0.01] | .08 | .003 | 0.01 | [0.01, 0.01] | .05 | .087 | 0.01 | [0.01, 0.01] | .05 | .079 |
| 1. Gender (Male = 0, Female = 1) | 0.30 | [0.20, 0.39] | .16 | < .001 | 0.16 | [0.11, 0.22] | .14 | < .001 | 0.41 | [0.32, 0.54] | .19 | < .001 |
| 1. Underlying health condition (No = 0, Yes = 1) | 0.30 | [0.19, 0.40] | .14 | < .001 | 0.09 | [0.02, 0.15] | .07 | .009 | 0.28 | [0.15, 0.40] | .12 | < .001 |
| 1. Frontline healthcare (No = 0, Yes = 1) | -0.07 | [-0.30, 0.17] | -.01 | .577 | -0.04 | [-0.18, 0.11] | -.01 | .615 | -0.11 | [-0.39, 0.16] | -.02 | .419 |
| 1. Education | -0.01 | [-0.07, 0.05] | -.01 | .770 | -0.03 | [-0.06, 0.01] | -.04 | .147 | -0.01 | [-0.07, 0.07] | -.01 | .908 |
| 1. USA = 0 vs UK = 1 | 0.14 | [0.03, 0.24] | .07 | .013 | -0.17 | [-0.23, -0.11] | -.14 | < .001 | -0.48 | [-0.07, 0.07] | -.20 | < .001 |
| 1. USA = 0 vs Remaining = 1 | -0.07 | [-0.21, 0.06] | -.03 | .285 | -0.01 | [-0.09, 0.08] | -.01 | .910 | -0.50 | [-0.07, 0.07] | -.17 | < .001 |
| *R*^2^ | .10 | | | | .06 | | | | .13 | | | |
| *F* | *F*(10, 1451) = 16.127* | | | | *F*(10, 1451) = 9.008* | | | | *F*(10, 1451) = 22.465* | | | |
| ⁎ *p* < .001. | | | | | | | | | | | | |

| Table S3  ***Study 2****: Suspected COVID-19 status moderation analyses (****controlling for nationality****).* | | | | | | | | |
| --- | --- | --- | --- | --- | --- | --- | --- | --- |
|  | Social Distancing | | | | | | | |
|  | Step 1 | | | | Step 2 | | | |
| Variable | *B* | 95% CI | β | *p* | *B* | 95% CI | β | *p* |
| 1. Suspected status | -0.13 | [-0.34, 0.08] | -.06 | .222 | -0.26 | [-0.49, -0.03] | -.12 | .025 |
| 1. Certainty | -0.08 | [-0.16, -0.01] | -.11 | .031 | -0.03 | [-0.11, 0.06] | -.03 | .001 |
| 1. Age | 0.01 | [0.01, 0.02] | .14 | .005 | 0.01 | [0.01, 0.02] | .14 | .003 |
| 1. Gender (Male = 0, Female = 1) | 0.18 | [0.02, 0.34] | .10 | .033 | 0.18 | [0.02, 0.34] | .11 | .024 |
| 1. Underlying health condition (No = 0, Yes = 1) | -0.34 | [-0.53, -0.16] | -.18 | < .001 | -0.34 | [-0.52, -0.16] | -.18 | < .001 |
| 1. Frontline healthcare (No = 0, Yes = 1) | -0.64 | [-1.06, -0.23] | -.14 | .003 | -0.59 | [-1.01, -0.07] | -.13 | .006 |
| 1. Education | -0.09 | [-0.19, 0.01] | -.09 | .063 | -0.09 | [-0.19, 0.01] | -.09 | .068 |
| 1. USA = 0 vs UK = 1 | 0.08 | [-0.10, 0.26] | .04 | .394 | 0.08 | [-0.10, 0.26] | .04 | .386 |
| 1. USA = 0 vs Remaining = 1 | -0.39 | [-0.62, -0.15] | -.16 | .001 | -0.40 | [-0.64, -0.17] | -.16 | .001 |
| 1. Suspect status X certainty |  |  |  |  | -0.25 | [-0.43, -0.07] | -.17 | .007 |
| *R*^2^ | .14 | | | | .15 | | | |
| *F* | *F*(9, 398) = 7.058** | | | | *F*(10, 397) = 7.199** | | | |
| *ΔR*^2^ |  | | | | .02 | | | |
| *ΔF* |  | | | | *F*(1, 397) = 7.445* | | | |
| ⁎⁎ *p* < .001, ⁎ *p* < .01. | | | | | | | | |


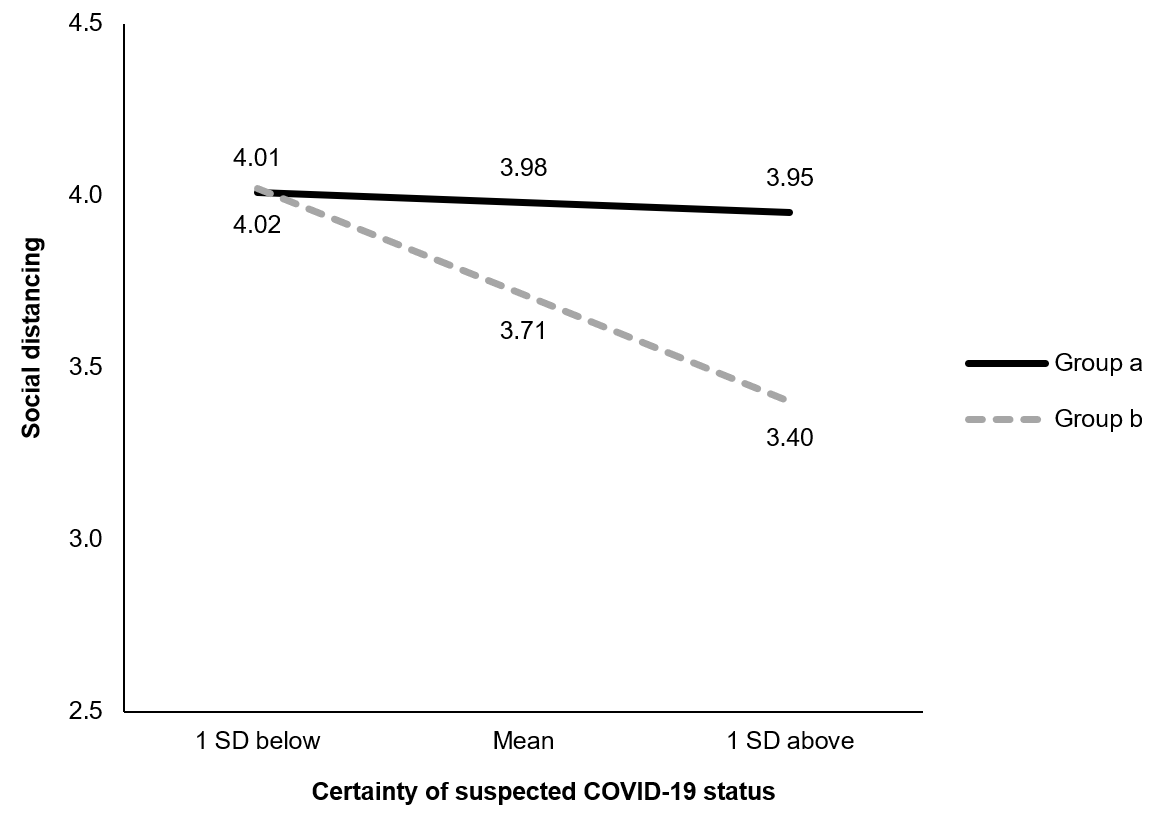


| **Figure S1**. **Study 2**: Moderating effect of certainty of suspected COVID-19 status on social distancing intentions. At low certainty, there is no difference in social distancing intentions between either suspected COVID-19 status groups. However, as certainty increases to mid and high levels, suspected recovery from COVID-19 (group *b*) reduces social distancing intentions, compared to people who do not suspect they were infected in the past (group *a;* ***controlling for nationality****).* |
| --- |

**Using group *d* as the control in experimental analyses**

Here were demonstrate that the main pattern of results in the experimental analyses remains the same even when the control group was replaced with only participants from group *d* (have been tested, results negative). These participants were under the same conditions as the control, but they were not originally included in the experimental randomized design. In Study 1, the control group originally consisted of participants from group *a* (not tested, do not suspect to be currently infected) only, and in Study 2 the control group originally consisted of participants from group *a* and *b* (not tested, do not suspect to be currently infected, but suspect to have had it in the past). The main pattern of results did not change when only group *d* was included in the control group for Study 1 (see Table S4) or Study 2 (see Table S5).

| Table S4  ***Study 1****: Predictors of social distancing and hygiene intentions over the next month, for the experimental conditions (****using group d [have been tested, results negative] as the control instead of group a [not tested, do not suspect to be currently infected]****).* | | | | | | | | |
| --- | --- | --- | --- | --- | --- | --- | --- | --- |
|  | Social distancing | | | | Hygiene | | | |
| Variable | *B* | 95% CI | β | *p* | *B* | 95% CI | β | *p* |
| 1. Recovered vs. control | -0.26 | [-0.43, -0.09] | -.16 | .002 | 0.01 | [-0.11, 0.12] | .01 | .970 |
| 1. Infected vs. control | 0.48 | [0.31, 0.65] | .30 | < .001 | 0.14 | [0.02, 0.25] | .14 | .018 |
| 1. Age | 0.01 | [-0.01, 0.01] | -.01 | .890 | 0.01 | [-0.01, 0.01] | .05 | .237 |
| 1. Gender (Male = 0, Female = 1) | 0.19 | [0.08, 0.29] | .11 | .001 | 0.14 | [0.06, 0.21] | .14 | < .001 |
| 1. Underlying health condition (No = 0, Yes = 1) | 0.13 | [0.02, 0.25] | .08 | .022 | 0.07 | [-0.01, 0.14] | .07 | .074 |
| 1. Frontline healthcare (No = 0, Yes = 1) | 0.01 | [-0.21, 0.24] | .01 | .912 | -0.07 | [-0.22, 0.08] | -.04 | .345 |
| 1. Education | 0.04 | [-0.03, 0.10] | .04 | .288 | 0.01 | [-0.04, 0.05] | .01 | .740 |
| *R*^2^ | .20 | | | | .04 | | | |
| *F* | *F*(7, 722) = 27.252* | | | | *F*(7, 722) = 5.129* | | | |
| ⁎ *p* < .001. | | | | | | | | |

| Table S5  ***Study 2****: Predictors of social distancing, hygiene, and face covering intentions over the next month, for the experimental conditions (****using group d [have been tested, results negative] as the control instead of group a [not tested, do not suspect to be currently infected]****).* | | | | | | | | | | | | |
| --- | --- | --- | --- | --- | --- | --- | --- | --- | --- | --- | --- | --- |
|  | Social distancing | | | | Hygiene | | | | Face covering | | | |
| Variable | *B* | 95% CI | β | *p* | *B* | 95% CI | β | *p* | *B* | 95% CI | β | *p* |
| 1. Immunity vs. control | -0.27 | [-0.44, -0.10] | -.13 | .002 | -0.08 | [-0.18, 0.02] | -.06 | .129 | -0.32 | [-0.52, -0.11] | -.13 | .002 |
| 1. Incautious vs. control | -0.34 | [-0.51, -0.18] | -.17 | < .001 | -0.04 | [-0.14, 0.06] | -.04 | .412 | -0.25 | [-0.45, -0.05] | -.10 | .013 |
| 1. Cautious vs. control | 0.06 | [-0.11, 0.22] | .03 | .510 | 0.01 | [-0.09, 0.11] | .01 | .865 | 0.01 | [-0.19, 0.21] | .01 | .920 |
| 1. Age | 0.01 | [-0.01, 0.01] | .04 | .134 | 0.01 | [0.01, 0.01] | .05 | .083 | 0.01 | [0.01, 0.01] | .06 | .041 |
| 1. Gender (Male = 0, Female = 1) | 0.35 | [0.25, 0.46] | .19 | < .001 | 0.19 | [0.13, 0.25] | .17 | < .001 | 0.46 | [0.34, 0.58] | .20 | < .001 |
| 1. Underlying health condition (No = 0, Yes = 1) | 0.29 | [0.17, 0.40] | .14 | < .001 | 0.10 | [0.03, 0.17] | .08 | .006 | 0.30 | [0.17, 0.44] | .12 | < .001 |
| 1. Frontline healthcare (No = 0, Yes = 1) | 0.13 | [-0.10, 0.35] | .03 | .268 | -0.07 | [-0.20, 0.07] | -.03 | .334 | 0.04 | [-0.23, 0.31] | .01 | .777 |
| 1. Education | 0.05 | [-0.04, 0.09] | .02 | .457 | -0.01 | [-0.04, 0.03] | -.01 | .757 | 0.01 | [-0.06, 0.09] | .01 | .790 |
| *R*^2^ | .11 | | | | .05 | | | | .09 | | | |
| *F* | *F*(8, 1217) = 18.000* | | | | *F*(8, 1217) = 7.875* | | | | *F*(8, 1217) = 15.303* | | | |
| ⁎ *p* < .001. | | | | | | | | | | | | |

**Excluding covariates from the main analyses**

| Table S6  ***Study 1****: Predictors of social distancing and hygiene intentions over the next month, for the experimental conditions (****all co-variates excluded****).* | | | | | | | | |
| --- | --- | --- | --- | --- | --- | --- | --- | --- |
|  | Social distancing | | | | Hygiene | | | |
| Variable | *B* | 95% CI | β | *p* | *B* | 95% CI | β | *p* |
| 1. Recovered vs. control | -0.12 | [-0.23, -0.02] | -.07 | .018 | 0.04 | [-0.04, 0.11] | .03 | .338 |
| 2. Infected vs. control | 0.59 | [0.49, 0.70] | .35 | < .001 | 0.15 | [0.08, 0.22] | .14 | < .001 |
| *R*^2^ | .15 | | | | .02 | | | |
| *F* | *F*(2, 1202) = 105.30* | | | | *F*(2, 1202) = 9.36* | | | |
| ⁎ *p* < .001. | | | | | | | | |

The remaining analyses demonstrate that the main pattern of results remain unchanged when covariates are excluded, for the experimental (Table S6 and S7) and moderation analyses.

| Table S7  ***Study 2****: Predictors of social distancing, hygiene, and face covering intentions over the next month, for the experimental conditions (****all co-variates excluded****).* | | | | | | | | | | | | |
| --- | --- | --- | --- | --- | --- | --- | --- | --- | --- | --- | --- | --- |
|  | Social distancing | | | | Hygiene | | | | Face covering | | | |
| Variable | *B* | 95% CI | β | *p* | *B* | 95% CI | β | *p* | *B* | 95% CI | β | *p* |
| 1. Immunity vs. control | -0.39 | [-0.50, -0.27] | -.18 | < .001 | 0.03 | [-0.04, 0.10] | .02 | .416 | -0.31 | [-0.45, -0.18] | -.13 | < .001 |
| 1. Incautious vs. control | -0.28 | [-0.40, -0.16] | -.13 | < .001 | 0.01 | [-0.07, 0.09] | .01 | .797 | -0.32 | [-0.46, -0.18] | -.13 | < .001 |
| 1. Cautious vs. control | 0.02 | [-0.10, 0.14] | .01 | .727 | 0.07 | [-0.01, 0.14] | .05 | .071 | -0.03 | [-0.16, 0.11] | -.01 | .714 |
| *R*^2^ | .04 | | | | .01 | | | | .02 | | | |
| *F* | *F*(3, 1752) = 21.93* | | | | *F*(3, 1752) = 1.24* | | | | *F*(3, 1752) = 12.15* | | | |
| ⁎ *p* < .001. | | | | | | | | | | | | |

***Suspected COVID-19 status (Study 1)***

We performed analyses testing whether *certainty of suspected COVID-19 status* moderated the effect of *suspected COVID-19 status* (0 = group *a* versus 1 = group *b*) on social distancing, hygiene and worry about COVID-19, but this time without the inclusion of covariates.

In Step 1, suspected COVID-19 status still did not predict social distancing intentions (β = -.07, 95% CI [-.23, .01], *p* = .051; *R*^2^ = .01, *F*(2, 848) = 3.44, *p* = .032), hygiene intentions (β = .01, 95% CI [-.07, .09], *p* = .747; *R*^2^ = .01, *F*(2, 848) = 0.18, *p* = .835), but now negatively predicted worry about COVID-19 (β = -.08, 95% CI [-.44, -.02], *p* = .029; *R*^2^ = .01, *F*(2, 855) = 5.15, *p* = .006). Certainty of suspected COVID-19 status now negatively predicted social distancing intentions (β = -.09, 95% CI [-.11, -.01], *p* = .019), but still negatively predicted worry about COVID-19 (β = -.11, 95% CI [-.24, -.05], *p* = .003), and not hygiene intentions (β = .01, 95% CI [-.03, .05], *p* = .555).

In Step 2, the interaction term still did not significantly account for increased variance for social distancing intentions (β = -.08, 95% CI [-.14, .07], *p* = .464; *ΔR*^2^ = .01, *ΔF*(3, 487) = 0.54, *p* = .464), hygiene intentions (β = .01, 95% CI [-.07, .07], *p* = .984; *ΔR*^2^ = .01, *ΔF*(3, 847) = 0.01, *p* = .984), or worry about COVID-19 (β = .15, 95% CI [-.04, .33], *p* = .123; *ΔR*^2^ = .01, *ΔF*(3, 854) = 2.39, *p* = .123).

***Suspected COVID-19 status (Study 2)***

As in Study 1, we performed analyses testing whether *certainty of suspected COVID-19 status* moderated the effect of *suspected COVID-19 status* (0 = group *a* versus 1 = group *b*) on social distancing, hygiene and face covering intentions, and worry about COVID-19, but this time without the inclusion of covariates.

In Step 1, suspected COVID-19 status still did not predict social distancing intentions (β = .07, 95% CI [-.05, .34], *p* = .132; *R*^2^ = .11, *F*(2, 484) = 3.04, *p* = .049), hygiene intentions (β = -.01, 95% CI [-.15, .12], *p* = .825; *R*^2^ = .01, *F*(2, 484) = 0.68, *p* = .508), face covering intentions (β = .03, 95% CI [-.14, .29], *p* = .493; *R*^2^ = .01, *F*(2, 484) = 3.66, *p* = .027), or worry about COVID-19 (β = .04, 95% CI [-.19, .50], *p* = .385; *R*^2^ = .01, *F*(2, 486) = 4.37, *p* = .013). Certainty of suspected COVID-19 status still negatively predicted social distancing intentions (β = -.11, 95% CI [-.16, -.01], *p* = .019), face covering intentions (β = -.11, 95% CI [-.18, -.03], *p* = .007), and worry about COVID-19 (β = -.19, 95% CI [-.32, -.06], *p* = .003), and did not predict hygiene intentions (β = .03, 95% CI [-.02, .08], *p* = .252).

In Step 2, the interaction term significantly accounted for increased variance for social distancing intentions (β = .18, 95% CI [.01, .34], *p* = .037; *ΔR*^2^ = .01, *ΔF*(3, 483) = 4.39, *p* = .037), and did not for hygiene intentions (β = .10, 95% CI [-.02, .21], *p* = .101; *ΔR*^2^ = .01, *ΔF*(3, 483) = 2.71, *p* = .101), face covering intentions (β = .04, 95% CI [-.14, .22], *p* = .652; *ΔR*^2^ = .01, *ΔF*(3, 483) = 2.50, *p* = .059), or worry about COVID-19 (β = .09, 95% CI [-.23, .37], *p* = .645; *ΔR*^2^ = .01, *ΔF*(3, 485) = 2.98, *p* = .031). Simple slopes analysis using PROCESS (Model 1, Hayes, 2003) revealed that the relationship between certainty of suspected COVID-19 status and social distancing intentions was negative and significant for group *b* (β = -.34, *p* < .001) but was not significant for group *a* (β = -.05, *p* = .366; Figure 1).
